# Relationships of depression and antidepressant use with epigenetic age acceleration and all-cause mortality among postmenopausal women

**DOI:** 10.18632/aging.205868

**Published:** 2024-05-27

**Authors:** May A. Beydoun, Hind A. Beydoun, Jason Ashe, Michael F. Georgescu, Steve Horvath, Ake Lu, Anthony S. Zannas, Aladdin H. Shadyab, Su Yon Jung, Sylvia Wassertheil-Smoller, Ramon Casanova, Alan B. Zonderman, Robert L. Brunner

**Affiliations:** 1 Laboratory of Epidemiology and Population Sciences, National Institute on Aging, NIA/NIH/IRP, Baltimore, MD 21224, USA; 2 VA National Center on Homelessness Among Veterans, U.S. Department of Veterans Affairs, Washington, DC 20420, USA; 3 Department of Management, Policy, and Community Health, School of Public Health, University of Texas Health Science Center at Houston, Houston, TX 77030, USA; 4 Department of Human Genetics, David Geffen School of Medicine, University of California Los Angeles, Los Angeles, CA 90095, USA; 5 Department of Biostatistics, School of Public Health, University of California Los Angeles, Los Angeles, CA 90095, USA; 6 Department of Psychiatry, School of Medicine, University of North Carolina at Chapel Hill, Chapel Hill, NC 27599, USA; 7 Herbert Wertheim School of Public Health and Human Longevity Science and Division of Geriatrics, Gerontology, and Palliative Care, Department of Medicine, University of California, San Diego, CA 92093, USA; 8 Department of Epidemiology, Fielding School of Public Health, Translational Sciences Section, School of Nursing, University of California, Los Angeles, CA 90095, USA; 9 Department of Epidemiology and Population Health, Albert Einstein College of Medicine, Bronx, NY 10461, USA; 10 Department of Biostatistics and Data Science, Wake Forest University School of Medicine, Winston-Salem, NC 27101, USA; 11 Department of Family and Community Medicine (Emeritus), School of Medicine, University of Nevada, Reno, NV 89557, USA

**Keywords:** depressive symptoms, epigenetic age acceleration, mortality, aging

## Abstract

We investigated relations of depressive symptoms, antidepressant use, and epigenetic age acceleration with all-cause mortality risk among postmenopausal women. Data were analyzed from ≤1,900 participants in the Women's Health Initiative study testing four-way decomposition models. After a median 20.4y follow-up, 1,161 deaths occurred. Approximately 11% had elevated depressive symptoms (EDS^+^), 7% were taking antidepressant medication at baseline (ANTIDEP^+^), while 16.5% fell into either category (EDS_ANTIDEP^+^). Baseline ANTIDEP^+^, longitudinal transition into ANTIDEP^+^ and accelerated epigenetic aging directly predicted increased mortality risk. GrimAge DNA methylation age acceleration (AgeAccelGrim) partially mediated total effects of baseline ANTIDEP^+^ and EDS_ANTIDEP^+^ on all-cause mortality risk in socio-demographic factors-adjusted models (Pure Indirect Effect >0, *P* < 0.05; Total Effect >0, *P* < 0.05). Thus, higher AgeAccelGrim partially explained the relationship between antidepressant use and increased all-cause mortality risk, though only prior to controlling for lifestyle and health-related factors. Antidepressant use and epigenetic age acceleration independently predicted increased all-cause mortality risk. Further studies are needed in varying populations.

## INTRODUCTION

Frequently under-recognized [1, 2], depression is a major contributor to the Global Burden of Diseases while being the most prevalent mental illness among geriatric populations [2]. Previous studies have established depression as a risk factor for age-related chronic conditions such as metabolic syndrome, diabetes mellitus, and cardiovascular disease [3]. By the same token, antidepressants are among the most widely prescribed medications in older adults [4]. Long-term health consequences of antidepressant use have not been adequately evaluated although a quarter of individuals prescribed antidepressants take them for ≥10 years [5]. Moreover, U.S. FDA guidance for long-term use of antidepressants relates mostly to Major Depressive Disorder (MDD), although a large percentage of antidepressant users do not meet the diagnostic criteria for MDD, and several classes of antidepressants are prescribed for other indications besides depression [6]. Although antidepressants can be effective at reducing depressive symptoms and potentially improving cognitive function and quality of life, they have been linked to side-effects such as weight gain, hyponatremia, reduced bone mineral density, tremor, sexual dysfunction, lessened general well-being, suicide, as well as increased risks of falls, fractures, and cardiovascular morbidities, with implications for compliance with prescribed treatments [4, 7]. As such, it is important to examine the separate and joint contributions of depression and antidepressant use to age-related health outcomes and their underlying processes.

Postmenopausal women constitute a high-risk group for both depression and antidepressant use since mental illnesses – including depression – increase with age and predominantly affect females [2]. Evidence from the Women’s Health Initiative (WHI) suggests that depression or antidepressant use may increase the vulnerability of postmenopausal women to age-related health problems including weight gain [8, 9], diabetes mellitus [10, 11], pre-hypertension and hypertension [12], cardiovascular disease [8, 13], cognitive dysfunction [4, 14], colorectal cancer [6], bone loss and fracture [15], hip and knee osteoarthritis [16], Parkinson’s disease [17], as well as frailty [7], with detrimental impact on cancer survivorship [18, 19], all-cause and cause-specific mortality [18–20] risks. Depression and antidepressants may be linked to health problems due to factors like inflammatory responses [21–24], neurotoxicity [25, 26], and epigenetic changes [27], with certain antidepressants potentially possessing anti-inflammatory properties [6].

Previous studies focused on health disparities have explored epigenetic age acceleration as a potential mediator for the effect of demographic (e.g., race), socioeconomic (e.g., education) and psychosocial factors – including depression – on morbidity and mortality risks, in general, and among postmenopausal women, in particular [28–30]. A mediator is often defined as an intermediate variable on the pathway between an exposure and an outcome that explains part of the effect of the exposure on that outcome variable. Accounting for this third variable often alters the total effect (TE) between the exposure and the outcome leading to an attenuation of the TE towards the null, a phenomenon known as consistent mediation [31–33]. In other instances, the TE becomes biased away from the null value, a phenomenon known as inconsistent mediation [31–33]. More generally, a mediator is influenced by the exposure and is on the causal pathway between the exposure and the outcome [31–33]. A third variable can be both a mediator and a moderator and can also be only a moderator or neither of the two. A moderator interacts with the exposure of interest to alter the TE of the exposure on the outcome in a way that the effect of the exposure on the outcome differs across levels of that third variable [31–33]. Although several recent studies have examined the association between depression and epigenetic age acceleration [34–39], few also examined the use of antidepressants [38, 39].

Epigenetic clocks are biomarkers that reflect biological aging based on DNA methylation (DNAm) of cytosine phosphate guanine (CpG) sites [28]. They are distinct from clinical and molecular markers that capture more limited aspects of aging [28]. Epigenetic clocks have been developed that predict longevity [29] and are strongly correlated with chronological age across distinct cell, tissue, and organ types [28, 40, 41]. Epigenetic clocks show promise in elucidating biological mechanisms pertaining to aging, chronic disease, and mortality risks [29]. Chronological age has been shown to increase levels of methylation in specific regions of the genome [29]. “DNAm age” – also known as “epigenetic age” – represents innate aging processes at the cellular level which have been linked to functional decline with age [30]. Epigenetic age can be estimated using multivariable regression models of DNAm profiles, and a discrepancy between DNAm age and chronological age, known as epigenetic age acceleration (EAA), has been associated with adverse health outcomes [28–30, 41]. A higher “DNAm age” compared to chronological age suggests faster biological aging than expected [30]. Epigenetic age acceleration is linked to obesity, early menopause, Down syndrome, Werner syndrome, HIV infection, lung cancer, Alzheimer’s and Parkinson’s diseases, and is determined partly by genetic factors and partly by environmental, psychosocial, and behavioral factors [40, 41]. Two epigenetic clocks, blood-based Hannum (71 CpGs) and pan-tissue Horvath (353 CpGs), can be used to derive extrinsic and intrinsic epigenetic age acceleration (EEAA and IEAA) by calculating the difference between DNAm and chronologic ages [28, 42]. Age-related changes in methylation of 353 CpGs included in the Horvath epigenetic clock, are known to influence DNA replication and repair, lipid metabolism, oxidative stress, and other processes linked to chronic diseases [42, 43], while epidemiologic evidence suggests that the Horvath estimator may predict cognitive function, lung function, physical strength, and premature mortality [42]. PhenoAge and GrimAge are next-generation epigenetic clocks from which EAA can also be estimated [29, 30].

Taken together, depression is a prevalent mental disorder among older populations, is linked to various diseases, particularly in postmenopausal women, and as a result may be influenced by epigenetic clocks. Furthermore, postmenopausal women are more likely than other groups to be prescribed antidepressant medications. Thus, depression (or elevated depressive symptoms, EDS) and/or anti-depressant use’s potential association with mortality risk may be mediated or potentially moderated by epigenetic clocks. Moreover, epigenetic clocks have been associated with increased mortality risk [44–46]. This positive association may be mediated by or moderated through depressive symptoms and/or through anti-depressant use. The interplay between elevated depressive symptoms (and/or anti-depressant use), epigenetic age acceleration and mortality risk remains largely unknown, particularly among postmenopausal women.

The present cohort study performed longitudinal analyses of existing observational data from the Women’s Health Initiative (WHI) ancillary study, to examine epigenetic age acceleration as a mediator/moderator between EDS (and/or antidepressant use) as a primary exposure of interest and all-cause mortality as the outcome among postmenopausal women. A secondary analysis was also conducted with EAA as the main exposure, all-cause mortality the outcome of interest and potential mediators/moderators being elevated depressive symptoms (EDS) and/or antidepressant use.

## MATERIALS AND METHODS

### Data source

The WHI is a long-term study focused on strategies for preventing heart disease, breast, and colorectal cancers as well as osteoporosis in postmenopausal women. The WHI study design, eligibility criteria, recruitment methods and measurement protocols are described elsewhere [47, 48]. Briefly, the WHI collected data on a multiethnic sample of postmenopausal women who were recruited and enrolled between 1993 and 1998 at 40 geographically diverse clinical centers (24 states and the District of Columbia) in the United States. The WHI study received institutional review board approval with informed consent from all participating clinical centers. WHI-Clinical Trials (CTs) (*n* = 68,132) and WHI-Observational Study (OS) (*n* = 93,676) are two components of the WHI (*n* = 161,808). Whereas WHI-CTs evaluated outcomes of menopausal hormone therapy (Hormone Therapy (HT) Trials), calcium and vitamin D supplementation ((CaD) Trial), and a low-fat eating pattern (Dietary Modification Trial), the WHI-OS evaluated causes of morbidity and mortality in postmenopausal women. The main WHI studies occurred between 1993 and 2005, and of 150,076 participants who were actively followed-up at the end of these studies, 76.9% participated in Extension Study 1 (2005–2010) and 86.9% of those eligible participated in Extension Study 2 (2010–2020) [49, 50]. At enrollment (1993–1998), WHI participants, 50–79 years of age, underwent a clinical examination and completed the same self-administered questionnaire covering demographics, general health, clinical and anthropometric characteristics, functional status, healthcare behaviors, reproductive, medical, and family history, personal habits, thoughts and feelings, therapeutic class of medication, hormones, supplements, and dietary intake, and several of these characteristics were assessed at later follow-up times.

### Study participants

We restricted this analysis to WHI participants with available DNAm data at enrollment (1993–1998) who took part in an ancillary case-control study (BA23) focused on identifying novel genomic determinants of coronary heart disease, as previously reported by others [30, 41]. In this integrative genomics study, cases and controls had already undergone genome-wide genotyping at baseline as well as profiling of seven cardiovascular biomarkers, with oversampling of African American and Hispanic participants [51]. Specifically, a stratified, racially/ethnically diverse sample of ≈ 2,200 WHI-CT participants with available stored serum were selected for analysis of DNA methylation at screening or annual visits [29]. At enrollment (1993–1998), blood samples were collected from participants, placed in EDTA tubes after an overnight fast, and stored at −80°C for processing by WHI core laboratories [29]. Patients with evidence of leukemia at enrollment (1993–1998) were excluded from these analysis [29]. Further details can be found under the following link: https://sp.whi.org/researchers/data/WHIStudies/StudySites/BA23/pages/home.aspx. In brief, this sub-study aimed at evaluating miRNA and methylation levels in coronary heart disease (CHD) events in 1070 patients and 1070 controls. Researchers used high-throughput genomic techniques to assess methylation status and miRNA levels in circulating white blood cells. They also used statistical techniques, machine learning, and sparse predictors to identify regulators and co-methylation modules linked to CHD. The study also reviewed genome-wide association studies to identify hundreds of molecular sub-phenotypes and CHD susceptibility polymorphisms.

Of the available 2,200 participants, 1,900 had complete data on baseline EDS and/or antidepressant use, and had known socio-demographics, particularly age, race, and ethnicity. All other demographic and socio-economic factors as well as lifestyle and health-related covariates were subjected to multiple imputation, as described later.

### DNA methylation

Illumina Infinium Human-Methylation 450 Bead Chip at the HudsonAlpha Institute of Biotechnology was used to perform analyses of DNA methylation [29]. Epigenetic clocks were calculated using genome-wide DNA methylation data that can estimate epigenetic age (DNAm) using the proportion of modified signal at each CpG site [29].

### Study variables

#### Elevated depressive symptoms, EDS

A depressive symptoms screening algorithm previously developed by Burnam et al. with scores ranging between 0 and 1 and higher scores consistent with greater burden of depressive symptoms were generated using 6 items from the 20-item CES-D scale and 2 items from the National Institute of Mental Health’s Diagnostic Interview Schedule. Furthermore, we dichotomized this variable based on a pre-established threshold of 0.06, whereby WHI participants with a score >0.06 have strong evidence of depressive symptoms whereas those with a score ≤0.06 do not [52, 53]. Repeated measures of EDS at enrollment (1993–1998) and 3-year follow-up were examined to evaluate change over time. Specifically, women were classified as having no change (0) if their status did not change between enrollment (1993–1998) and 3-year follow-up time, an increase (or transition into EDS^+^) (+) if they were non-depressed at enrollment (1993–1998) and depressed at 3-year follow-up time or a decrease (or transition out of EDS^−^) (−) if they were depressed at enrollment (1993–1998) and non-depressed at 3-year follow-up.

#### Antidepressant use, ANTIDEP

WHI participants were instructed to bring prescription and non-prescription medication containers at enrollment (1993–1998). For medications used for >2 weeks, drug names and doses were entered into a medications database and assigned therapeutic class codes using the Master Drug Data Base (MDDB: Medi-Span, Indianapolis, IN; Medi-Span software: First DataBank, Inc., San Bruno, CA, USA). Antidepressant use at enrollment was defined as a dichotomous (‘yes’ or ‘no’) variable (form 44) based on the following therapeutic class codes used for WHI at baseline and at 3-years of follow-up: α-2 receptor antagonists (Tetracyclics) (580300), MAO inhibitors (581000), modified cyclics (581200), selective serotonin reuptake inhibitors (581600), serotonin-norepinephrine reuptake inhibitors (581800), tricyclic agents (582000), miscellaneous antidepressants (583000), antidepressant combinations (589900, 589980, 589985, 589987, 589990). Repeated measures of antidepressant use at enrollment (1993–1998) and 3-year follow-up were examined to evaluate change over time. Specifically, women were classified as having no change (0) if their status did not change between enrollment (1993–1998) and 3-year follow-up time, an increase (or transition into ANTIDEP^+^) (+) if they were non-users of antidepressants at enrollment (1993–1998) and users of antidepressants at 3-year follow-up time or a decrease (i.e. transition out of ANITIDEP^+^) (−) if they were users of antidepressants at enrollment (1993–1998) and non-users of antidepressants at 3-year follow-up. Due to sample size limitations, we combined all types of antidepressants for the main analyses.

#### EDS and/or antidepressant use

A categorical variable was defined by combining the dichotomous variables for EDS and antidepressant use (ANTIDEP) as follows: [1] no EDS and no antidepressant use; [2] no EDS and antidepressant use; [3] EDS and no antidepressant use; [4] EDS and antidepressant use. This variable was used to describe the interaction between depression and antidepressant use. Due to sample size limitations, a dichotomous version of depression and/or antidepressant use was defined for use in this analysis to compare women who had EDS or were antidepressant users to those who were neither depressed nor antidepressant users at enrollment (1993–1998) or 3 years of follow-up. Repeated measures of EDS and/or antidepressant use at enrollment (1993–1998) and 3-year follow-up was examined to evaluate change over time. Specifically, women were classified as having no change (0) if their status did not change between enrollment (1993–1998) and 3-year follow-up time, an increase or a transition into EDS^+^ or ANTIDEP^+^ (+) if they were either EDS^−^ or non-users of antidepressants at enrollment (1993–1998) and either EDS^+^ or users of antidepressants at 3-year follow-up time or a decrease or transition out of EDS^+^ or ANTIDEP^+^ (−) if they were EDS^+^ or users of antidepressants at enrollment (1993–1998) and EDS^−^ or non-users of antidepressants at 3-year follow-up.

#### Epigenetic age acceleration

We defined epigenetic age acceleration using residuals from regression of biological age on chronological age, with biological age defined according to four distinct epigenetic clocks, namely the blood-based Hannum (EEAA) and pan-tissue Horvath (IEAA) estimators described previously as well as next-generation estimators called PhenoAge and GrimAge yielding AgeAccelPheno and AgeAccelGrim, respectively. These four measures of epigenetic age acceleration have been previously calculated in the context of BA23 and are available for further analyses.

#### Extrinsic epigenetic age acceleration (EEAA)

The EEAA is based on 71 CpGs as well as counts of naïve and exhausted cytotoxic T cells, and plasma B cells, which are known to be associated with age in order to calculate Hannum’s estimator of “DNAm age” [29, 30]. In sum, it is a weighted average of “DNAm age” and WBC cell composition that vary with age. In this study, we used residual values of EEAA from a regression model involving “weighted DNAm age” in relation to “chronological age” [29, 30].

#### Intrinsic epigenetic age acceleration (IEAA)

IEAA is based on 353 CpGs used to calculate Horvath’s estimator for “DNAm age” [30]. In this study, we relied on an estimate of IEAA calculated as a residual from regressing “DNAm age” on “chronological age” and estimated measures of WBC cell composition (naïve and exhausted CD8+ T cells, CD4+ T cells, plasma B cells, natural killer cells, monocytes, and granulocytes) to adjust for confounding by WBC changes in composition occurring with aging [29, 30].

#### AgeAccelPheno

This next-generation measure of epigenetic age acceleration is based on the “PhenoAge” estimator derived from an algorithm that comprises 513 CpGs and that can predict chronological age as well as a composite of physiological indicators such as albumin and glucose [29, 30]. *AgeAccelPheno* was estimated using the residual method from a model with Age as the outcome.

#### AgeAccelGrim

This next-generation measure of epigenetic age acceleration is based on the “GrimAge” estimator derived in two stages. At the first stage, surrogates of pack-years of smoking and 12 plasma proteins were defined based on “DNAm age”. At the second stage, time-to-death was regressed on these surrogates. “GrimAge” epigenetic clock consists of 1,030 CpG sites that jointly predict mortality risk [29, 30]. *AgeAccelGrim* was estimated using the residual method from a model with Age as the outcome.

#### Summary of the EAA measures

Epigenetic age acceleration is defined by utilizing residuals from regression of biological age on chronological age, using four distinct epigenetic clocks: blood-based Hannum (EEAA), pan-tissue Horvath (IEAA), PhenoAge, and GrimAge. These four measures of epigenetic age acceleration have been previously calculated in the context of BA23 and are available for further analyses. Extrinsic epigenetic age acceleration (EEAA) is based on 71 CpGs and counts of naïve and exhausted cytotoxic T cells and plasma B cells, which are known to be associated with age. Intrinsic epigenetic age acceleration (IEAA) is based on 353 CpGs and is used to calculate Horvath’s estimator for “DNAm age.” AgeAccelPheno is a next-generation measure of epigenetic age acceleration based on the “PhenoAge” estimator, which can predict chronological age and a composite of physiological indicators such as albumin and glucose. AgeAccelGrim is based on the “GrimAge” estimator, which consists of 1,030 CpG sites that jointly predict mortality risk.

### All-cause mortality risk

Deaths were ascertained via semi-annual or annual follow-up with family, friends, and medical care providers of WHI participants, in addition to the National Death Index and obituaries [54]. In this study, participants were followed up through December 31st, 2021, to evaluate all-cause mortality risks. Censoring age for those who did not experience the event of interest was age at baseline plus the number of years for the longest follow-up time, given that follow-up ended in December 31st, 2021. Survival time was set up using baseline age for time of entry, age at event or censoring for time of exit (end of follow-up or loss to follow-up) and event (yes vs. no) for all-cause mortality, with the origin being baseline age.

### Covariates

The hypothesized relationships are potentially confounded by socio-demographic, lifestyle and health characteristics that are known to be associated with mortality risks and may be related to EDS and/or antidepressant use. Covariates collected at the enrollment visit included BA23 case-control status, socio-demographic characteristics (race (American Indian/Alaska Native, Asian, Native Hawaiian/Other Pacific Islanders, Black, White, More than one race, Unknown/Not reported), recoded into “White”, “Black” and “Others” (missing were excluded); ethnicity (Hispanic, non-Hispanic, Unknown/Not reported), education (less than high school, high school, some college, completed college or higher level), household income (<$20,000, $20,000–$49,999, $50,000–$99,999, ≥$100,000), marital status (Married/Partnered, Single, Divorced, Widowed)), lifestyle characteristics (smoking status (Never Smoker, Past Smoker, Current Smoker), alcohol consumption (Non-Drinker, Former Drinker, <1 drink/week, ≥1 drink/week), physical activity (Metabolic equivalent-hours/week)), and health characteristics, namely, body mass index (BMI), comorbid conditions (cardiovascular disease (Yes, No), hypertension (Yes, No), hyperlipidemia (Yes, No), diabetes (Yes, No)), and self-rated health (Excellent/Very Good/Good, Fair/Poor)). Trained staff collected anthropometric data, including weight (kg) and height [55] at enrollment [56]. Weight was measured to the nearest 0.1 kg on a balance beam scale with the participant dressed in indoor clothing without shoes, while height was measured to the nearest 0.1 cm using a wall-mounted stadiometer. BMI was calculated as (weight (kg) ÷ (height^2^ (m^2^)) and further categorized as <25.0 kg/m^2^ (underweight/normal weight); 25.0–29.9 kg/m^2^ (overweight); and ≥30 kg/m^2^ (obese). The history of cardiovascular disease was defined in terms of previous coronary heart disease, angina, aortic aneurysm, carotid endarterectomy or angioplasty, atrial fibrillation, congestive heart failure, cardiac arrest, stroke, or transient ischemic attack. The history of hypertension was defined as self-reported diagnosis or treatment for hypertension or evidence of high blood pressure based on systolic blood pressure (SBP) and diastolic blood pressure (DBP) measurements. The history of diabetes was defined as physician-diagnosed diabetes or use of diabetes medications. The history of hyperlipidemia was defined as using lipid-lowering medications or having been told of high cholesterol by a physician.

### Statistical analysis

All statistical analyses were conducted using SAS version 9.4 (SAS Institute, Cary, NC, USA) for data management and STATA version 18 (StataCorp, College Station, TX, USA) for bivariate and multivariable analyses.

First, summary statistics included mean ± standard errors for continuous variables and frequencies with percentages for categorical variables. Kaplan-Meier estimates for survival probabilities were plotted against the 3 main baseline exposures of interest (baseline EDS, antidepressant use (ANTIDEP) and the combined baseline exposure EDS_ANTIDEP) across follow-up time (years) from baseline age to exit through censoring or event. Log-rank tests were conducted and other survival time descriptives (years) are reported along with exposure prevalence estimates.

Second, simple and multivariable linear, multinomial logistic or Cox PH regression models were constructed to estimate β±SE, Log_e_ of the odds ratios (OR) or hazard ratios (HR) with their SE, respectively. We examined the bivariate association of baseline socio-demographic, lifestyle, and health characteristics with several alternative outcomes, namely epigenetic age acceleration, EDS and/or antidepressant use, as well as all-cause mortality risks. We further constructed binary and multinomial logistic regression models to examine the relationship of epigenetic age acceleration with EDS, antidepressant use as well as EDS and/or antidepressant use (defined as categorical and dichotomous variables) at enrollment (1993–1998) and 3-year follow-up.

Third, we constructed Cox regression models to examine the relationships of epigenetic age acceleration, EDS, antidepressant use as well as EDS and/or antidepressant use (defined as categorical and dichotomous variables) at enrollment (1993–1998) and 3-year follow-up with all-cause mortality risks, before and after adjustment of covariates.

Fourth, we applied causal mediation analyses to examine the mediating and/or moderating effects of epigenetic age acceleration on the relationship of EDS and antidepressant use at enrollment (1993–1998) (and the combination of the two), with mortality risk. Specifically, *med4way* STATA command was used to estimate the mediating and/or moderating effect of z-transformed epigenetic age acceleration scores on the TE of EDS and/or antidepressant use on mortality risk, controlling for baseline characteristics [57–59].

This causal mediation analysis is helpful in a counterfactual framework and in the context of observational data whereby two models are estimated, namely, a model for the mediator conditional on exposure and covariates and a model for the outcome conditional on the exposure, mediator, and covariates [57–59]. Assuming no unmeasured confounding and 4-way decomposition, the *med4way* command can also facilitate estimation of mediation but not interaction (pure indirect effect (PIE)), interaction but not mediation (reference interaction (INTREF)), both mediation and interaction (mediated interaction (INTMED)) and neither mediation nor interaction (controlled direct effect (CDE)), whereby the TE can be calculated as follows: TE = CDE+PIE+INTEF+INTMED [57]. Four-way decomposition parameters were estimated on the multiple-imputed data using Rubin’s rule to obtain averages of these estimates along with their Standard Errors and *p*-values. Percentages of TE accounted for by each of these four components were also estimated, though without SE or *p*-value, which cannot be directly estimated from multiple-imputed data. Thus, the statistical significance of the PIE in models where TE was statistically significant at a type I error of 0.05 and where PIE and TE have the same direction, determined consistent mediation. The degree to which TE was mediated with the third variable was determined by the percent PIE of TE.

Three alternative models were tested, by incrementally including exogenous covariates: Model 1 (unadjusted), Model 2 (adjusted only for socio-demographic variables, including measures of socio-economic status) and Model 3 (Model 2 further adjusted for lifestyle and health-related factors).

Fifth, to study bi-directional associations, another set of four-way decomposition models were conducted, whereby EDS and/or antidepressant use were considered as alternative mediators, while epigenetic age acceleration measures were the main exposures of interest assumed to have a positive TE on mortality risk. In this final set of models, the mediator equation was a logistic regression model given that all the alternative mediators were binary.

Given the limited sample size, we performed multiple imputations (5 datasets, 10 iterations) of covariates after selecting the sample of interest, based on inclusion and exclusion criteria, as described by Lee and Carlin [60]. Two-sided statistical tests were conducted at α = 0.05. Supplementary Figure 1 shows a graphical depiction of the study design, focusing on the time frame of measures for exposure, mediator and outcome as well as exclusion criteria.

## RESULTS

**Figure 1:**
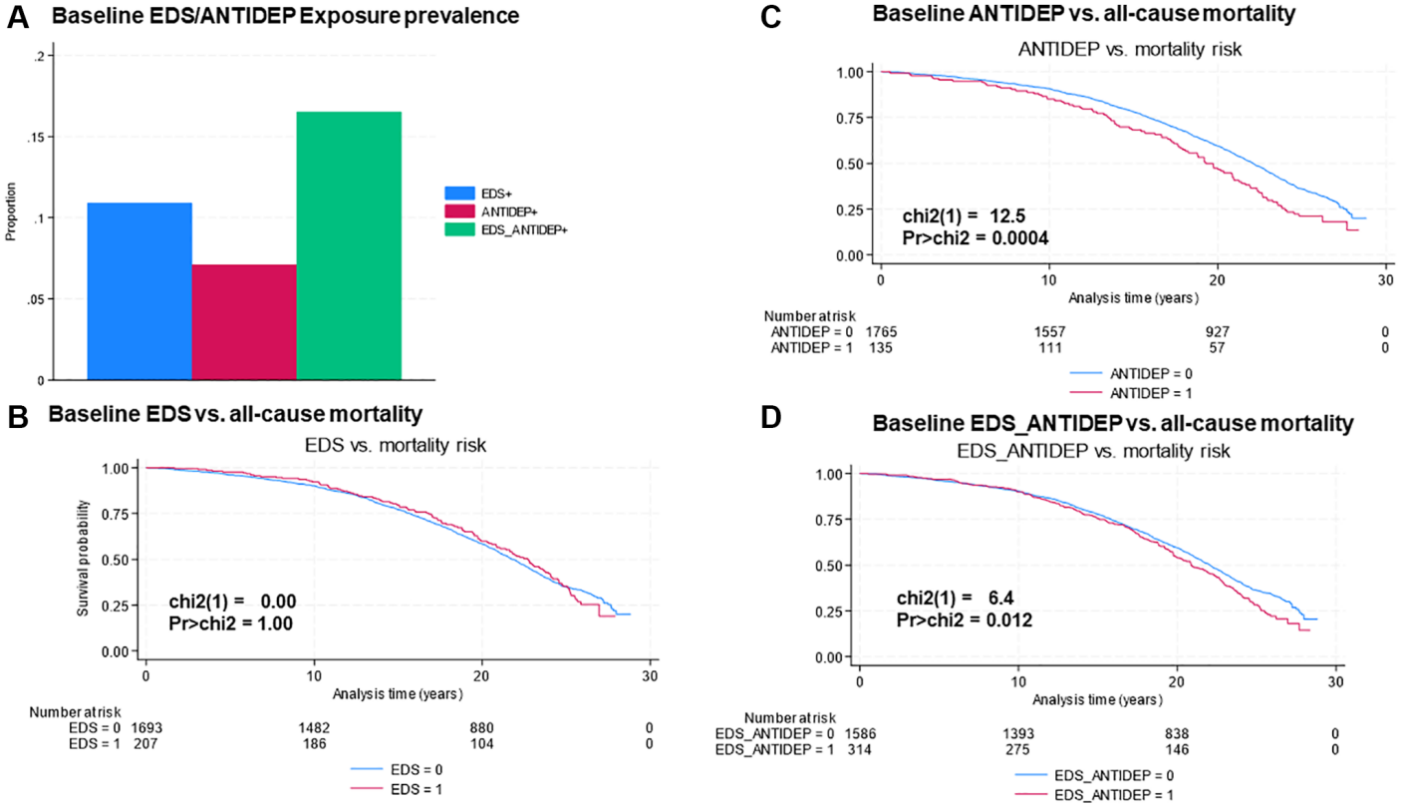
(**A**–**D**) Elevated depressive symptoms and antidepressant use baseline exposures vs. all-cause mortality risk, Women’s Health Initiative Study. Abbreviations: EDS: Elevated depressive symptoms; ANTIDEP: Antidepressant use; EDS_ANTIDEP: Elevated depressive symptoms and/or antidepressant use.

After a mean time of follow-up of 20.4 years (range: 0.10–28.8 years), 1,161 deaths occurred in the largest selected sample of 1,900 postmenopausal women. Thus, median survival time was 21.9 years with an interquartile range of 15.7–27.48 years, and incidence rate was estimated at 3,247 per 100,000 P-Y (output and Stata script provided on GitHub at https://github.com/baydounm/WHI_EPIDGENETICCLOCK_DEP_MORT). With proportions of 11–17% (Figure 1D), the associations of the 3 baseline exposures (EDS/ANTIDEP) with all-cause mortality risk is presented in Figure 1A–1C, using Kaplan-Meier (K-M) survival probability estimates and log-rank tests. Overall, only baseline antidepressant use (ANTIDEP) and the combined exposure (EDS_ANTIDEP) were significantly associated with increased all-cause mortality risk in this sample (Log-rank test, 1 d.f. (ANTIDEP): 12.5, *P* = 0.0004; Figure 1B; Log-rank test, 1 d.f. (EDS_ANTIDEP): 6.4, *P* = 0.012; Figure 1C), while mortality risk was comparable between EDS^+^ and EDS^−^ groups.

Table 1 displays study sample characteristics, overall and their relationship with the four epigenetic age acceleration metrics. The mean ± SE of baseline age was 64.6 ± 0.2 y, 32% of the sample consisted of Black adults, while 65.7% were White. Hispanic ethnicity was self-reported by 14.8% of this sample. Over half (53%) were married/partnered, with 29.5% being college graduates and 30.0% with incomes below $20,000. In terms of lifestyle and health-related factors, 53.3% were never smokers, 15.4% were non-drinkers, Met-hour/week was on average 10.1, while body mass index was 29.8 kg.m^−2^, with obesity screened for 43.9% of the sample. History of cardiovascular disease, hypertension, diabetes, and dyslipidemia was reported by 17.6%, 53.3%, 23.5% and 16.3%, respectively. Around 11% of the sample had EDS at baseline, while 12.5% reported their health as fair/poor. Bivariate differences in baseline characteristics were found in terms of epigenetic age acceleration metrics, with consistently faster acceleration with respect to hypertension and BMI, across all four metrics, and in the case of diabetes for 3 of 4 metrics. Most notably, and unlike all other epigenetic clocks under study, GrimAge age acceleration was increased by +0.78y when comparing those with EDS vs. not at baseline (*P* < 0.010), a pattern also observed for self-rated health (fair/poor vs. excellent/very good/good: +0.86 y, *p* < 0.010). It is worth noting that EAA metrics differed by race, though without a consistent pattern that was discerned.

**Table 1:** Associations of sociodemographic, lifestyle and health characteristics with epigenetic age acceleration (*n* = 1,900) – Women’s Health Initiative.

	**Overall**	**IEAA**	**EEAA**	**AgeAccel Pheno**	**AgeAccel GrimAge**
**Total:**	% or mean ± SE	+0.04 ± 0.12	−0.23 ± 0.15	−0.15 ± 0.15	−0.06 ± 0.09
		SD = 5.2	SD = 6.5	SD = 6.5	SD = 3.9
***Age (years):***					
Continuous	64.6 ± 0.2	−0.002 ± 0.02	+0.012 ± 0.021	+0.006 ± 0.021	−0.003 ± 0.012
50–54	25.9	Ref	Ref	Ref	Ref
55–69	47.0	+0.19 ± 0.28	−0.04 ± 0.36	+0.65 ± 0.36	+0.18 ± 0.22
70–79+	27.1	−0.14 ± 0.32	0.16 ± 0.40	+0.16 ± 0.41	+0.15 ± 0.24
***Race:***					
White	65.7	Ref	Ref	Ref	Ref
Black	32.4	+0.04 ± 0.25	−1.73 ± 0.31^***^	+0.93 ± 0.32^**^	+1.35 ± 0.19^***^
Other	1.9	−1.83 ± 0.86^*^	−0.07 ± 1.07	+0.52 ± 1.09	+1.38 ± 0.64^*^
***Ethnicity:***					
Non-Hispanic	85.2	Ref	Ref	Ref	Ref
Hispanic	14.8	−1.19 ± 0.33^***^	+2.14 ± 0.41^***^	+0.35 ± 0.42	−0.87 ± 0.25^***^
***Marital status:***					
Married/Partnered	53.0	Ref	Ref	Ref	Ref
Single	4.2	−0.29 ± 0.60	−0.97 ± 0.76	−1.28 ± 0.75	+0.36 ± 0.44
Divorced	19.5	−0.14 ± 0.31	−0.30 ± 0.39	+0.61 ± 0.39	+1.26 ± 0.23^***^
Widowed	23.3	−0.52 ± 0.29	+0.27 ± 0.36	+0.12 ± 0.37	+0.92 ± 0.22^***^
***Education:***					
Less than high school	11.1	Ref	Ref	Ref	Ref
High school graduate	20.0	+0.48 ± 0.44	−0.51 ± 0.55	−0.68 ± 0.57	−0.72 ± 0.33^*^
Some college	39.5	+0.56 ± 0.40	−1.07 ± 0.50^*^	−0.95 ± 0.51	−0.43 ± 0.30
College graduate	29.5	+0.54 ± 0.41	−2.04 ± 0.51^***^	−1.75 ± 0.52^**^	−1.23 ± 0.31^***^
***Household income:***					
<$20,000	30.0	Ref	Ref	Ref	Ref
$20,000–$49,999	47.2	+0.29 ± 0.27	−0.49 ± 0.35	−0.81 ± 0.36^*^	−0.51 ± 0.21^*^
$50,000–$99,999	18.4	+0.28 ± 0.35	−1.27 ± 0.44^**^	−1.12 ± 0.45^*^	−0.84 ± 0.27^**^
>$100,000	4.4	−0.02 ± 0.60	−1.89 ± 0.78^*^	−1.39 ± 0.78	−1.40 ± 0.46^**^
***Smoking status:***					
Never	53.3	Ref	Ref	Ref	Ref
Past	36.7	−0.17 ± 0.25	−0.54 ± 0.32	+0.24 ± 0.32	+2.14 ± 0.16^***^
Current	10.0	+0.07 ± 0.40	−0.23 ± 0.51	+1.60 ± 0.51^**^	+6.82 ± 0.26^***^
***Alcohol use:***					
Non-drinker	15.4	Ref	Ref	Ref	Ref
Former drinker	23.5	+0.50 ± 0.19	+0.51 ± 0.48	+0.38 ± 0.49	+1.78 ± 0.29^***^
<1 drink/week	33.8	+0.51 ± 0.36	+0.04 ± 0.46	−0.03 ± 0.46	+0.69 ± 0.27^*^
≥1 drink/week	27.3	+0.25 ± 0.37	−0.09 ± 0.47	−0.47 ± 0.47	+1.01 ± 0.28^***^
***Physical activity (Met-hours/week):***					
Continuous	10.1 ± 0.3	−0.008 ± 0.009	−0.023 ± 0.012^*^	−0.020 ± 0.012	−0.023 ± 0.007^**^
***Body Mass Index (kg/m^2^):***					
Continuous	29.8 ± 0.1	+0.049 ± 0.019^**^	+0.062 ± 0.024^**^	+0.119 ± 0.024^***^	+0.051 ± 0.014^***^
<25	22.6	Ref	Ref	Ref	Ref
25–29.9	33.5	−0.22 ± 0.32	−0.26 ± 0.40	+0.12 ± 0.40	−0.02 ± 0.24
≥30	43.9	+0.52 ± 0.30	+0.62 ± 0.38	+1.30 ± 0.38^**^	+0.54 ± 0.13^*^
***Medical history:***					
*Cardiovascular disease:*					
No	82.7	Ref	Ref	Ref	Ref
Yes	17.6	+0.51 ± 0.31	−0.23 ± 0.39	+0.81 ± 0.39^*^	+0.34 ± 0.23
*Hypertension:*					
No	46.7	Ref	Ref	Ref	Ref
Yes	53.3	+0.60 ± 0.23^*^	+0.58 ± 0.29^*^	+1.56 ± 0.30^***^	+0.75 ± 0.18^***^
*Diabetes:*					
No	76.5	Ref	Ref	Ref	Ref
Yes	23.5	+0.64 ± 0.27^*^	+0.41 ± 0.35	+1.33 ± 0.35^***^	+0.78 ± 0.21^***^
*Hyperlipidemia:*					
No	83.7	Ref	Ref	Ref	Ref
Yes	16.3	+0.19 ± 0.33	+0.22 ± 0.40	−0.05 ± 0.42	-0.09 ± 0.25
***EDS at baseline:***					
No	89.1	Ref	Ref	Ref	Ref
Yes	10.9	−0.38 ± 0.37	+0.80 ± 0.47	+0.34 ± 0.48	+0.78 ± 0.28^**^
***Self-rated health:***					
Excellent/Very good/Good	87.5	Ref	Ref	Ref	Ref
Fair/Poor	12.5	+0.45 ± 0.35	+0.90 ± 0.44	+0.84 ± 0.45	+0.86 ± 0.27^**^

Abbreviations: AgeAccel GrimAge: GrimAge epigenetic age acceleration; AgeAccel Pheno: PhenoAge epigenetic age acceleration; EDS: Elevated Depressive Symptoms; EEAA: Extrinsic Epigenetic Age Acceleration; IEAA: Intrinsic Epigenetic Age Acceleration; Met: Metabolic Equivalent; SD: Standard Deviation; SE: Standard Error; WHI: Women’s Health Initiative. Values are means ± SE or percentages for the overall sample; β coefficients ± SE from ordinary least square (OLS) bivariate models with outcome being each of 4 epigenetic clock metrics. Analysis was done on multiple imputed data. ^*^P < 0.05; ^**^P < 0.010; ^***^P < 0.001 for null hypothesis that β = 0. Bolded values are when *P* < 0.05.

Table 2 presents the results of the bivariate associations between baseline characteristics and the key outcomes or mediators, namely all-cause mortality (using Cox PH models) and EDS/antidepressant use at baseline (using a series of bivariate logistic regression models). The odds of EDS or combination of EDS and antidepressant use was reduced with advancing age, with higher education and income, greater physical activity, and was increased among current smokers, with higher BMI, and among those reporting fair/poor health. The latter also predicted antidepressant use, which was less prevalent among Black participants compared to their White counterparts. All-cause mortality (1,161 deaths by end of follow-up), on the other hand, was increased with age, reduced among Black vs. White participants, as well as among Hispanic vs. non-Hispanic participants, increased among single and widowed participants compared to their married/partnered counterparts, reduced with educational attainment and income levels, as well as physical activity, increased among current smokers, and in the presence of cardiovascular disease, hypertension, diabetes, hyperlipidemia and with a self-rated health as fair/poor (vs. excellent/very good/good).

**Table 2:** Associations of sociodemographic, lifestyle and health characteristics with baseline elevated depressive symptoms (EDS), antidepression use and all-cause mortality risk (*n* = 1,900) – Women’s Health Initiative.

	**Mortality**	**EDS/Antidepressant**		
	**Log_e_ (HR) ± SE 1,161 deaths, median follow-up: 20.4 y**	**EDS 11%**	**Antidepressant use, ANTIDEP 7.1%**	**EDS and/or Antidepressant, EDS_ANTIDEP 16.5%**
		**Log_e_ (OR) ± SE**	**Log_e_ (OR) ± SE**	**Log_e_ (OR) ± SE**
***Age (years):***				
Continuous	+0.104 ± 0.005^***^	−0.040 ± 0.010^***^	−0.007 ± 0.013	−0.022 ± 0.009^*^
50–54	Ref	Ref	Ref	Ref
55–69	+1.04 ± 0.096^***^	−0.439 ± 0.169^**^	+0.027 ± 0.218	−0.225 ± 0.146
70–79+	+1.86 ± 0.10^***^	−0.619 ± 0.202^**^	−0.045 ± 0.248	−0.319 ± 0.170
***Race***				
White	Ref	Ref	Ref	Ref
Black	−0.018 ± 0.065^**^	+0.150 ± 0.156	−0.763 ± 0.224**	−0.242 ± 0.137
Other	−0.032 ± 0.216	+0.749 ± 0.431	−1.200 ± 1.020	+0.127 ± 0.428
***Ethnicity***				
Non-Hispanic	Ref	Ref	Ref	Ref
Hispanic	−0.582 ± 0.099^***^	+0.467 ± 0.185^*^	+0.177 ± 0.239	+0.347 ± 0.161^*^
***Marital status:***				
Married/Partnered	Ref	Ref	Ref	Ref
Single	+0.288 ± 0.145^*^	+0.222 ± 0.370	−1.036 ± 0.727	−0.218 ± 0.350
Divorced	+0.029 ± 0.082	+0.317 ± 0.191	+0.396 ± 0.216	+0.254 ± 0.160
Widowed	+0.617 ± 0.069^**^	+0.334 ± 0.179	−0.0360 ± 0.230	+0.165 ± 0.152
***Education:***				
Less than high school	Ref	Ref	Ref	Ref
High school graduate	−0.139 ± 0.110	−0.616 ± 0.241^*^	+0.473 ± 0.362	−0.307 ± 0.213
Some college	−0.174 ± 0.100	−0.640 ± 0.213^**^	+0.435 ± 0.338	−0.372 ± 0.193
College graduate	−0.414 ± 0.106^***^	−0.996 ± 0.237^***^	0.157 ± 0.357	−0.649 ± 0.207^**^
***Household income:***				
<$20,000	Ref	Ref	Ref	Ref
$20,000–$49,999	−0.242 ± 0.068^*^	−0.889 ± 0.166^***^	−0.162 ± 0.206	−0.595 ± 0.140^***^
$50,000–$99,999	−0.587 ± 0.097^***^	−0.770 ± 0.219^***^	−0.382 ± 0.276	−0.626 ± 0.186^**^
>$100,000	−1.001 ± 0.186^***^	−1.560 ± 0.580^**^	−0.482 ± 0.526	−1.193 ± 0.436^**^
***Smoking status:***				
Never	Ref	Ref	Ref	Ref
Past	+0.021 ± 0.064	+0.024 ± 0.166	+0.317 ± 0.193	+0.176 ± 0.137
Current	+0.261 ± 0.097^**^	+0.721 ± 0.217^**^	+0.436 ± 0.288	+0.662 ± 0.192^**^
***Alcohol use:***				
Non-drinker	Ref	Ref	Ref	Ref
Former drinker	+0.082 ± 0.096	+0.328 ± 0.231	−0.153 ± 0.277	+0.254 ± 0.196
<1 drink/week	−0.142 ± 0.092	−0.013 ± 0.228	−0.300 ± 0.264	−0.045 ± 0.190
≥1 drink/week	−0.109 ± 0.094	−0.409 ± 0.250	−0.283 ± 0.275	−0.308 ± 0.204
***Physical activity (Met-hours/week):***
Continuous	−0.005 ± 0.003^*^	−0.025 ± 0.008^**^	−0.015 ± 0.008	−0.019 ± 0.006^**^
***Body Mass Index (kg/m^2^):***				
Continuous	+0.0001 ± 0.005	+0.038 ± 0.011^**^	+0.016 ± 0.014	+0.025 ± 0.010^*^
<25	Ref	Ref	Ref	Ref
25–29.9	−0.212 ± 0.079^**^	+0.104 ± 0.224	+0.196 ± 0.249	+0.146 ± 0.178
≥30	+0.074 ± 0.075	+0.588 ± 0.203^**^	+0.143 ± −0.240	+0.367 ± 0.166^*^
***Medical history:***				
*Cardiovascular disease:*				
No	Ref	Ref	Ref	Ref
Yes	+0.317 ± 0.074^***^	+0.225 ± 0.185	+0.145 ± 0.227	+0.200 ± 0.157
*Hypertension:*				
No	Ref	Ref	Ref	Ref
Yes	+0.592 ± 0.061^***^	+0.214 ± 0.149	+0.132 ± 0.180	+0.228 ± 0.125
*Diabetes:*				
No	Ref	Ref	Ref	Ref
Yes	+0.164 ± 0.067^*^	+0.211 ± 0.167	+0.261 ± 0.200	+0.247 ± 0.140
*Hyperlipidemia:*				
No	Ref	Ref	Ref	Ref
Yes	+0.304 ± 0.078^***^	+0.066 ± 0.201	+0.069 ± 0.245	+0.084 ± 0.169
***Self-rated health:***				
Excellent/Very good/Good	Ref	Ref	Ref	Ref
Fair/Poor	+0.329 ± 0.087^***^	1.309 ± 0.171^***^	+0.615 ± 0.227^**^	**+1.062 ± 0.155**

Abbreviations: ANTIDEP: Antidepressant use; CI: Confidence Interval; EDS: Elevated Depressive Symptoms; EDS_ANTIDEP: Either EDS or ANTIDEP; Met: Metabolic Equivalent; OR: Odds Ratio; WHI: Women’s Health Initiative. Values are Log_e_ of hazard ratios (HR) with SE or Log_e_ of odds ratios (OR) with SE, from Cox PH and logistic regression models, respectively. ^*^P < 0.05; ^**^P < 0.010; ^***^P < 0.001 that β = 0 or Log_e_ (HR) = 0. Bolded values are when *P* < 0.05.

The relationship between epigenetic age acceleration metrics and EDS/antidepressant use measures is presented in Table 3, using a series of unadjusted and multivariable-adjusted logistic regression models. Among the four metrics, only AgeAccelGrim was associated with antidepressant use or the combination of EDS and antidepressant use, in both the unadjusted and the partially adjusted model for sociodemographic variables, including age, race, ethnicity, marital status, education and income. In contrast, antidepressant use or the combination of EDS and antidepressant use were no longer associated with AgeAccelGrim, upon further adjustment for lifestyle and health-related factors.

**Table 3:** Logistic regression models for elevated depressive symptoms (EDS) and/or antidepressant use at baseline, as predicted by estimates of epigenetic age acceleration (*n* = 1,900)^a^.

	**Model 1**	**Model 2**	**Model 3**
	**Log_e_ (OR) ± SE**	**Log_e_ (OR) ± SE**	**Log_e_ (OR) ± SE**
*EDS:*			
IEAA	−0.015 ± 0.015 *P* = 0.31	−0.010 ± 0.015 *P* = 0.51	−0.016 ± 0.015 *P* = 0.28
EEAA	***+0.020 ± 0.012 P = 0.089***	+0.014 ± 0.012 *P* = 0.25	+0.008 ± 0.012 *P* = 0.52
AgeAccelPheno	+0.008 ± 0.011 *P* = 0.48	+0.003 ± 0.116 *P* = 0.79	−0.002 ± 0.012 *P* = 0.84
AgeAccelGrim	**+0.050 ± 0.018 *P* = 0.006**	**+0.042 ± 0.019 *P* = 0.029**	+0.008 ± 0.024 *P* = 0.75
*Antidepressant use, ANTIDEP:*			
EEAA	+0.016 ± 0.014 *P* = 0.23	+0.011 ± 0.015 *P* = 0.47	+0.007 ± 0.015 *P* = 0.62
IEAA	+0.017 ± 0.017 *P* = 0.32	+0.016 ± 0.018 *P* = 0.36	+0.014 ± 0.018 *P* = 0.42
AgeAccelPheno	−0.004 ± 0.014 *P* = 0.76	−0.004 ± 0.015 *P* = 0.80	−0.011 ± 0.015 *P* = 0.46
AgeAccelGrim	**+0.045 ± 0.022 *P* = 0.045**	**+0.057 ± 0.023 *P* = 0.014**	+0.037 ± 0.029 *P* = 0.20
*EDS and/or antidepressant use, EDS_ANTIDEP:*			
IEAA	+0.0004 ± 0.0122 *P* = 0.97	+0.003 ± 0.0125 *P* = 0.83	−0.002 ± 0.013 *P* = 0.86
EEAA	+0.0142 ± 0.0097 *P* = 0.14	+0.007 ± 0.010 *P* = 0.47	+0.002 ± 0.010 *P* = 0.84
AgeAccelPheno	+0.0016 ± 0.0104 *P* = 0.87	−0.001 ± 0.010 *P* = 0.90	−0.008 ± 0.010 *P* = 0.43
AgeAccelGrim	**+0.0434 ± 0.0156 *P* = 0.005**	**+0.044 ± 0.016 *P* = 0.007**	+0.008 ± 0.020 *P* = 0.71

Abbreviations: AgeAccel GrimAge: GrimAge epigenetic age acceleration; AgeAccel Pheno: PhenoAge epigenetic age acceleration; ANTIDEP: Antidepressant use; EDS: Elevated Depressive Symptoms; EDS_ANTIDEP: Either EDS or ANTIDEP; EEAA: Extrinsic Epigenetic Age Acceleration; IEAA: Intrinsic Epigenetic Age Acceleration; OR: Odds Ratio; SE: Standard Error; WHI: Women’s Health Initiative. ^a^Values are Log_e_ of Odd Ratios (OR) with SE from multiple logistic regression models on multiple imputed data. Main outcome are alternative measures of baseline depression, antidepressant use or a combination of the two (and/or) at baseline. Exposures are four epigenetic clock metrics, untransformed. Model 1 is unadjusted; Model 2 is adjusted for sociodemographic factors; Model 3 is Model 2 further adjusted for lifestyle and health characteristics.

Table 4 shows the main findings from a series of Cox PH models with incremental adjustment for covariates, examining the associations between epigenetic age acceleration metrics, EDS/antidepressant use exposures and the all-cause mortality outcome. In all models, EEAA, AgeAccelPheno, and AgeAccelGrim were associated with increased risk of mortality, suggesting that those metrics were predictive of mortality risk independently of sociodemographic, lifestyle, and health-related factors. In contrast, IEAA was only associated with all-cause mortality in the unadjusted and socio-demographic factor-adjusted models. While baseline EDS exposure was not associated with all-cause mortality, baseline antidepressant use and combined baseline EDS and antidepressant use were associated with this outcome in the unadjusted and socio-demographic factor-adjusted model, whereas antidepressant use was significantly associated with higher mortality risk after further adjustment for lifestyle and health-related factors. Furthermore, increased use of antidepressants between baseline and 3-year follow-up compared to no change in use was linked to increased mortality risk in all three models, with some attenuation between models 2 and 3 (Log_e_HR = +0.52 ± 0.15, *P* < 0.001 in Model 2 vs. Log_e_HR = +0.42 ± 0.15 in Model 3, *P* < 0.010). No association was found between change in EDS status between baseline and 3-year follow-up and mortality or combined change in EDS/antidepressant use between those two waves of data. It is worth noting that sample sizes differed among the 3 dynamic exposures (antidepressant use change: *n* = 1,696; EDS status change: *n* = 473; combined EDS and anti-depressant use: *n* = 462).

**Table 4:** Cox proportional hazard models for baseline and change in elevated depressive symptom status (EDS status), antidepressant use (ANTIDEP), EDS and/or antidepressant use (EDS_ANTIDEP) as well as epigenetic age acceleration predictors of all-cause mortality risk (*n* = 1,900).

	**Model 1**	**Model 2**	**Model 3**
	**Log_e_ (HR) ± SE**	**Log_e_ (HR) ± SE**	**Log_e_ (HR) ± SE**
**EPIGENETIC AGE ACCELERATION, per year:**			
IEAA	+0.006 ± 0.006	+0.012 ± 0.006^*^	+0.008 ± 0.006
EEAA	+0.017 ± 0.005^***^	+0.020 ± 0.005^***^	+0.017 ± 0.005^***^
AgeAccelPheno	+0.026 ± 0.005^***^	+0.028 ± 0.005^***^	+0.023 ± +0.005^***^
AgeAccelGrim	+0.0676 ± 0.0073^***^	+0.074 ± 0.008^***^	+0.061 ± 0.010^***^
**EDS AND/OR ANTIDEPRESSANT USE:**			
***BASELINE:***			
*EDS:*			
Yes vs. No, 11%	+0.000 ± 0.095	+0.057 ± 0.097	−0.069 ± 0.099
*Antidepressant use, ANTIDEP:*			
Yes vs. No, 7.1%	+0.372 ± 0.106^***^	+0.431 ± 0.107^***^	+0.362 ± 0.109^***^
*EDS and/or antidepressant use, EDS_ANTIDEP:*			
Yes vs. No, 16.5%	+0.193 ± 0.077^*^	+0.234 ± 0.077^**^	+0.132 ± 0.080
***CHANGE:***			
*EDS, N = 473*			
Increase vs. No change, 8.2%	−0.231 ± 0.252	−0.255 ± 0.261	−0.387 ± 0.267
Decrease vs. No change, 5.7%	−0.119 ± 0.267	+0.002 ± 0.278	−0.008 ± 0.287
*Antidepressant use, ANTIDEP, N = 1,696*			
Increase vs. No change, 4.1%	+0.464 ± 0.144^***^	+0.524 ± 0.145^***^	+0.424 ± 0.147^**^
Decrease vs. No change, 0.0%	n/a	n/a	n/a
*EDS and/or antidepressant use, EDS_ANTIDEP, N = 462*			
Increase vs. No change, 6.9%	+0080 ± 0.240	+0.229 ± 0.252	+0.372 ± 0.264
Decrease vs. No change, 9.5%	+0.030 ± 0.220	−0.039 ± 0.225	−0.151 ± 0.231

Abbreviations: AgeAccel GrimAge: GrimAge epigenetic age acceleration; AgeAccel Pheno: PhenoAge epigenetic age acceleration; ANTIDEP: Antidepressant use; EDS: Elevated Depressive Symptoms; EDS_ANTIDEP: Either EDS or ANTIDEP; EEAA: Extrinsic Epigenetic Age Acceleration; IEAA: Intrinsic Epigenetic Age Acceleration; HR: Hazard Ratio; SE: Standard Error. Values are Log_e_ of Hazard Ratios (HR) with standard error (SE) from multiple Cox proportional hazards model of all-cause mortality, with main exposures being each of the epigenetic clock metrics (untransformed), and each of the depression and/or antidepressant metrics. Model 1 is unadjusted; Model 2 is adjusted for sociodemographic factors; Model 3 is Model 2 further adjusted for lifestyle and health characteristics. Model 1 is unadjusted; Model 2 adjusted for sociodemographic; Model 3 is Model 2 further adjusted for lifestyle and health characteristics. Total deaths for the 3 CHANGE samples were: 254 for change in EDS (*N* = 473), 1,014 for change in antidepressant use (*N* = 1,696) and 246 for change in either EDS or antidepressant use (*N* = 462). ^*^P < 0.05; ^**^P < 0.010; ^***^P < 0.001 for null hypothesis that Log_e_ (HR) = 0. Bolded values are when *P* < 0.05.

Causal mediation using four-way decomposition models was carried out on the baseline exposures and potential mediators/moderators in relation to all-cause mortality risk. In Table 5, the three exposures were baseline EDS and antidepressant exposures (EDS (yes vs. no); antidepressant use (yes vs. no); and EDS and/or antidepressant use (yes vs. no)), the mediators were the four epigenetic age acceleration metrics. The models were tested, unadjusted for exogenous variables (Model 1), adjusted for socio-demographic covariates (Model 2), and the fully adjusted model that added lifestyle and health-related covariates to Model 2 (Model 3). TEs were statistically significant for both antidepressant use and EDS and/or antidepressant use exposures. Those were largely controlled direct effects (CDE) in Models 1 and 2. Nevertheless, a small proportion of this TE (TE >0, *P* < 0.05) was explained by AgeAccelGrim, most notably 10.5% of the TE in Model 2 for the antidepressant use exposure (PIE >0, *P* < 0.05), and 19.7% of the TE (TE >0, *P* < 0.05; PIE >0, *P* < 0.05) in Model 2 for the combined exposure at baseline (i.e., EDS_ANTIDEP). In contrast, when all exogenous variables were adjusted for in Model 3, only the TE of the antidepressant use variable remained statistically significant and was not mediated or moderated by any of the epigenetic age acceleration metrics, and therefore was mainly composed of a CDE.

**Table 5:** Causal mediation analysis (four-way decomposition models) of the total effects of baseline elevated depressive symptoms (EDS), antidepressant use (ANTIDEP) and combined exposure (EDS_ANTIDEP) on mortality risk with epigenetic age acceleration measures as alternative mediators and/or moderators (*n* = 1,900).

**M**	**X**	**Four-way decomposition parameter**	**MODEL 1**			**MODEL 2**			**MODEL 3**		
			**β**	**SE**	***P***	**β**	**SE**	**P**	**β**	**SE**	***P***
IEAA	EDS	TE	0.0017106	0.095751	0.986	0.057647	0.102676	0.574	−0.05545	0.094308	0.557
IEAA	EDS	CDE	−0.0009915	0.095451	0.992	0.0567	0.102338	0.58	−0.066	0.09232	0.475
IEAA	EDS	INTREF	−0.0003691	0.004162	0.929	−0.00173	0.006309	0.784	0.002178	0.00998	0.827
IEAA	EDS	INTMED	0.0056475	0.010026	0.573	0.006152	0.010888	0.572	0.012847	0.014419	0.373
IEAA	EDS	PIE	−2.58E-03	0.003413	0.45	−3.48E-03	0.005387	0.519	−4.47E-03	0.004667	0.338
EEAA	EDS	TE	−0.0006863	0.095779	0.994	0.049619	0.10193	0.626	−0.07021	0.09239	0.447
EEAA	EDS	CDE	-0.0131615	0.095165	0.89	0.048497	0.102664	0.637	−0.06518	0.09304	0.484
EEAA	EDS	INTREF	−0.0006583	0.009407	0.944	−0.00492	0.007303	0.5	−0.00579	0.004247	0.173
EEAA	EDS	INTMED	−0.0009921	0.012992	0.939	−0.00475	0.01055	0.653	−0.00491	0.008971	0.585
EEAA	EDS	PIE	0.0141256	0.009243	0.126	0.010792	0.009831	0.272	0.005665	0.008845	0.522
AgeAccelPheno	EDS	TE	0.0000886	0.096919	0.999	0.048942	0.102393	0.633	−0.06832	0.092412	0.46
AgeAccelPheno	EDS	CDE	−0.0113608	0.094191	0.904	0.052792	0.101469	0.603	−0.06007	0.092736	0.517
AgeAccelPheno	EDS	INTREF	0.0021367	0.019468	0.913	−0.00675	0.017977	0.707	−0.00626	0.012055	0.604
AgeAccelPheno	EDS	INTMED	0.0006401	0.006157	0.917	−0.00073	0.00375	0.846	0.001089	0.004606	0.813
AgeAccelPheno	EDS	PIE	0.0086726	0.012413	0.485	0.00363	0.014195	0.798	−0.00307	0.011403	0.787
AgeAccelGrim	EDS	TE	−0.0266901	0.09377	0.776	0.046077	0.102604	0.653	−0.04957	0.095579	0.604
AgeAccelGrim	EDS	CDE	−0.0337597	0.092664	0.716	0.031192	0.10046	0.756	−0.03554	0.096764	0.713
AgeAccelGrim	EDS	INTREF	***−0.0228723***	***0.01333***	***0.086***	−0.01885	0.018102	0.298	***−0.01554***	***0.00875***	***0.076***
AgeAccelGrim	EDS	INTMED	−0.0271204	0.021055	0.198	−0.01486	0.018235	0.415	−0.00193	0.008711	0.825
AgeAccelGrim	EDS	PIE	**0.0570623**	**0.022187**	**0.01**	**0.048594**	**0.02296**	**0.034**	0.003433	0.015426	0.824
IEAA	ANTIDEP	TE	**0.452231**	**0.153859**	**0.003**	**0.538803**	**0.164389**	**0.001**	**0.440586**	**0.156856**	**0.005**
IEAA	ANTIDEP	CDE	**0.4542324**	**0.155238**	**0.003**	**0.537047**	**0.165304**	**0.001**	**0.442942**	**0.157676**	**0.005**
IEAA	ANTIDEP	INTREF	0.0001341	0.004183	0.974	−0.00125	0.003635	0.731	−0.0002	0.00492	0.968
IEAA	ANTIDEP	INTMED	−0.004634	0.016258	0.776	−0.00179	0.013365	0.894	−0.00503	0.013007	0.699
IEAA	ANTIDEP	PIE	0.0024985	0.00371	0.501	0.004791	0.005987	0.424	0.002868	0.004371	0.512
EEAA	ANTIDEP	TE	**0.4954547**	**0.164074**	**0.003**	**0.572487**	**0.171995**	**0.001**	**0.437305**	**0.156729**	**0.005**
EEAA	ANTIDEP	CDE	**0.4298175**	**0.152623**	**0.005**	**0.525322**	**0.164307**	**0.001**	**0.428169**	**0.156024**	**0.006**
EEAA	ANTIDEP	INTREF	0.0297936	0.038358	0.437	0.026051	0.038127	0.494	0.001755	0.015975	0.913
EEAA	ANTIDEP	INTMED	0.0246407	0.029007	0.396	0.013565	0.022883	0.553	0.002075	0.009315	0.824
EEAA	ANTIDEP	PIE	0.0112029	0.009952	0.26	0.007549	0.010763	0.483	0.005307	0.009532	0.578
AgeAccelGrim	ANTIDEP	TE	**0.4358111**	**0.152299**	**0.004**	**0.525749**	**0.164139**	**0.001**	**0.431893**	**0.155802**	**0.006**
AgeAccelGrim	ANTIDEP	CDE	**0.4495079**	**0.151611**	**0.003**	**0.538851**	**0.162376**	**0.001**	**0.446032**	**0.155677**	**0.004**
AgeAccelGrim	ANTIDEP	INTREF	−0.0104798	0.013628	0.442	−0.00932	0.018215	0.609	−0.00876	0.011756	0.456
AgeAccelGrim	ANTIDEP	INTMED	0.0016019	0.006686	0.811	0.000567	0.00425	0.894	0.003058	0.009643	0.751
AgeAccelGrim	ANTIDEP	PIE	−0.0048189	0.015498	0.756	−0.00435	0.0173	0.801	−0.00844	0.014017	0.547
AgeAccelGrim	ANTIDEP	TE	**0.4427156**	**0.155681**	**0.004**	**0.566686**	**0.174617**	**0.001**	**0.442176**	**0.158689**	**0.005**
AgeAccelGrim	ANTIDEP	CDE	**0.3828099**	**0.148107**	**0.01**	**0.440543**	**0.156218**	**0.005**	**0.414911**	**0.157088**	**0.008**
AgeAccelGrim	ANTIDEP	INTREF	0.0021218	0.026714	0.937	0.026911	0.037671	0.475	−0.00057	0.016763	0.973
AgeAccelGrim	ANTIDEP	INTMED	0.0104902	0.024429	0.668	0.039127	0.037188	0.293	0.004146	0.014253	0.771
AgeAccelGrim	ANTIDEP	PIE	***0.0472937***	***0.02474***	***0.056***	**0.060105**	**0.026512**	**0.023**	0.023691	0.018298	0.195
IEAA	EDS_ANTIDEP	TE	**0.2138826**	**0.09324**	**0.022**	**0.262237**	**0.097907**	**0.007**	0.147103	0.091558	0.108
IEAA	EDS_ANTIDEP	CDE	**0.2142893**	**0.093159**	**0.021**	**0.264621**	**0.097837**	**0.007**	0.146103	0.09096	0.108
IEAA	EDS_ANTIDEP	INTREF	−0.0003589	0.002716	0.895	−0.00203	0.004074	0.618	0.000081	0.0061	0.989
IEAA	EDS_ANTIDEP	INTMED	−0.0001182	0.003628	0.974	−0.00126	0.006324	0.841	0.001688	0.007677	0.826
IEAA	EDS_ANTIDEP	PIE	0.0000704	0.002159	0.974	0.000913	0.004477	0.838	−0.00077	0.003465	0.825
EEAA	EDS_ANTIDEP	TE	**0.2145101**	**0.093706**	**0.022**	**0.257794**	**0.097669**	**0.008**	0.135865	0.090505	0.133
EEAA	EDS_ANTIDEP	CDE	**0.20317**	**0.092624**	**0.028**	**0.257856**	**0.097592**	**0.008**	0.141998	0.09062	0.117
EEAA	EDS_ANTIDEP	INTREF	0.0000545	0.00869	0.995	−0.00422	0.00767	0.582	−0.00643	0.004583	0.161
EEAA	EDS_ANTIDEP	INTMED	0.0011981	0.009059	0.895	−0.00148	0.005139	0.773	−0.00156	0.006445	0.809
EEAA	EDS_ANTIDEP	PIE	0.0100875	0.007507	0.179	0.005645	0.008045	0.483	0.001857	0.007479	0.804
AgeAccelGrim	EDS_ANTIDEP	TE	**0.2044953**	**0.09317**	**0.028**	**0.24704**	**0.097143**	**0.011**	0.13677	0.090514	0.131
AgeAccelGrim	EDS_ANTIDEP	CDE	**0.208944**	**0.091911**	**0.023**	**0.260852**	**0.09639**	**0.007**	***0.15115***	***0.09056***	***0.095***
AgeAccelGrim	EDS_ANTIDEP	INTREF	−0.0059607	0.012763	0.64	−0.01296	0.012284	0.291	−0.01061	0.009004	0.239
AgeAccelGrim	EDS_ANTIDEP	INTMED	−0.0002728	0.001951	0.889	0.000461	0.004399	0.917	0.003837	0.006843	0.575
AgeAccelGrim	EDS_ANTIDEP	PIE	0.0017847	0.010754	0.868	−0.00131	0.012394	0.916	−0.00761	0.010221	0.457
AgeAccelGrim	EDS_ANTIDEP	TE	**0.1868112**	**0.09243**	**0.043**	**0.247715**	**0.098647**	**0.012**	0.146775	0.09202	0.111
AgeAccelGrim	EDS_ANTIDEP	CDE	**0.1647262**	**0.089639**	**0.066**	**0.209912**	**0.094102**	**0.026**	***0.15814***	***0.09245***	***0.087***
AgeAccelGrim	EDS_ANTIDEP	INTREF	−0.0151191	0.013719	0.27	−0.009	0.017296	0.603	***−0.01431***	***0.00868***	***0.099***
AgeAccelGrim	EDS_ANTIDEP	INTMED	−0.0106622	0.015063	0.479	−0.00257	0.015721	0.87	−0.0021	0.005776	0.716
AgeAccelGrim	EDS_ANTIDEP	PIE	**0.0478663**	**0.018415**	**0.009**	**0.049371**	**0.019335**	**0.011**	0.005045	0.013287	0.704

Abbreviations: AgeAccel GrimAge: GrimAge epigenetic age acceleration; AgeAccel Pheno: PhenoAge epigenetic age acceleration; ANTIDEP: Antidepressant use; CDE: Controlled Direct Effect; EDS: Elevated Depressive Symptoms; EDS_ANTIDEP: Either EDS or ANTIDEP; EEAA: Extrinsic Epigenetic Age Acceleration; HR: Hazard Ratio; IEAA: Intrinsic Epigenetic Age Acceleration; INTMED: Mediated Interaction; INTREF: Interaction Referent; PIE: Pure Indirect Effect; SE: Standard Error; TE: Total Effect; X: Exposure. Values are estimates ± SE from four-way decomposition models and their *p*-values, with final equation being a Cox PH model for all-cause mortality, exposure being each of the depressive symptoms and antidepressant use baseline exposures; and mediators being each of the epigenetic clock metrics. Model 1 is unadjusted; Model 2 adjusted for sociodemographic; Model 3 is Model 2 further adjusted for lifestyle and health characteristics. P is for null hypothesis that β = 0. Bolded values are when *P* < 0.05.

In Supplementary Table 1, a similar modeling strategy was carried out, though in this instance, epigenetic age acceleration metrics were the main exposures of interest while EDS/antidepressant use were the key potential mediators/moderators. In these models, TEs were generally indicative of a positive association between epigenetic age acceleration and mortality risk, even after adjustment for all potentially confounding variables. In all these models, TEs were mainly composed of controlled direct effects, suggesting that EDS, ANTIDEP and EDS_ANTIDEP at baseline did not mediate or moderate the association between epigenetic age acceleration and mortality risk.

## DISCUSSION

### Summary of findings

The study examined the impact of depressive symptoms, antidepressant use, and epigenetic age acceleration on all-cause mortality in postmenopausal women. Data from 1,900 participants were used to test associations between key exposures and outcomes. Bi-directional four-way decomposition models were conducted to examine the mediating and moderating roles of epigenetic age acceleration, EDS, and antidepressant use at baseline. After a median 20.4 years follow-up time, 1,161 deaths occurred. Around 11% had elevated depressive symptoms (EDS^+^), 7% were taking antidepressant medication at baseline (ANTIDEP^+^), while 16.5% fell into either category (EDS_ANTIDEP^+^). Baseline ANTIDEP^+^, longitudinal increase in ANTIDEP^+^ and accelerated epigenetic age acceleration directly predicted all-cause mortality risk. GrimAge age acceleration mediated part of the TEs of baseline ANTIDEP^+^ and EDS_ANTIDEP^+^ on all-cause mortality risk in sociodemographic factors-adjusted models, thus only prior to controlling for lifestyle and health-related factors (PIE >0, *P* < 0.05; TE >0, *P* < 0.05).

### Previous studies

Epigenetic age acceleration has been connected to both MDD and depression symptoms. For instance, Han et al. used data from the Netherlands Study of Depression and Anxiety (NESDA) with a standard cut point (14) on the Inventory of Depressive Symptomology and a follow up of 4 years. They found significantly higher epigenetic age acceleration in patients with MDD (*n* = 319 compared to controls *n* = 811) [61]. Although no additional associations between increased epigenetic age acceleration and cumulative clinical features were found, their data suggested that more advanced epigenetic age acceleration in MDD may be substantially explained by severity of depression [61]. In a recent analysis of the Healthy Aging of Neighborhoods of Diversity Across the Life Span (HANDLS) project, we found a cross-sectional relationship between two epigenetic age acceleration measures (the Horvath 1 and the Hannum clocks) and lower levels of positive affect only in White participants, which remained statistically significant after further adjusted for potential confounders, including in the case of the Hannum clock all socio-demographic and socio-economic factors in addition to health-related and/or dietary factors [34]. A case-control study with 60 age-matched controls and 49 MDD cases found a link between MDD and GrimAge acceleration [62]. GrimAge acceleration is a DNAm-based epigenetic clock which is an estimator of smoking pack-years and proxy DNAm biomarkers of seven different plasma proteins [62]. After adjusting for sex, current smoking status, and BMI, the link between GrimAge and MDD remained significant (*p* = 0.015) [62]. Using data from the Health and Retirement Study, GrimAge DNAm age was linked to long-term, persistently higher depressive symptoms [63]. Nevertheless, when accounting for smoking and BMI this connection was considerably diminished, becoming marginally significant when comparing high vs. low depressive symptom levels in the fully adjusted model [63]. This is in line with the earlier study with HANDLS data [34], in which no link was detected between depressive symptoms and epigenetic aging using the Horvath and Hannum epigenetic clocks, and only a weak association with the reduced positive affect component of depressive symptom measurement. Our present results are consistent with these previous findings.

It is well established that depression is a major risk factor predictive of all-cause mortality for adults across the life span. However, additional consideration for older adults remains a public health concern, given their vulnerabilities to psychosocial difficulties, such as loneliness and social isolation that may have a role in the diagnosis and/or its severity. In several longitudinal studies, higher mortality rates were observed among older adults living with more depressive symptoms, clinically diagnosed minor and major depression, and history of mood disorders compared to nondepressed adults [64, 65]. Prior studies have established these associations in populations outside of the U.S. [66, 67] and in samples previously diagnosed with chronic health conditions such as cancer or diabetes [18, 19]. Emerging work has shown modest or null associations between depression or depressive symptoms and all-cause mortality rates among older adult women [18, 19, 68]. Depression was related to all-cause mortality in postmenopausal women from China [68]. Other studies using U.S.-based female populations exclusively included women previously diagnosed with other comorbidities and terminal conditions such as breast and colorectal cancer and showed minimal effects [18, 19]. In one study using participant data from the WHI, women who developed depression before a breast cancer diagnosis, unlike women with a prior history of depression, showed a modestly heightened risk for mortality [19]. This finding suggests that developing a mood disorder later in life along with other severe illnesses might increase risk, but the nuances of these mechanisms are still unclear. Prior depression or depressive symptoms appeared to be strong predictors of all-cause mortality or death from breast cancer in this sample. However, irrespective of how depression or EDS were conceptualized, neither depressive symptoms nor clinically significant depression influenced the risk of death among women with colorectal cancer [18]. Though depression often remains a robust predictor of death from any cause for older adults, chronic diseases and health behaviors may be meaningful contributors as well [64, 65].

A primary surmised mechanism underlying mood disorder symptomatology and mortality risk proposes that for individuals living with minor, moderate, or major depression, the potential for hypothalamic-pituitary-adrenal (HPA) axis dysregulation is exacerbated, wherein neuroendocrine and inflammatory modulation can affect overall biological functioning and wellness [69]. Nevertheless, our study did not indicate that depression by itself was associated with all-cause mortality. In contrast, antidepressant use was an independent predictor of all-cause mortality, even after adjusting for key potential confounders including lifestyle and health-related factors. This coupled with persistent depressive symptoms or an increase in symptoms and medications over time simultaneously were also linked to all-cause mortality independently of measured risk factors. Evidence from the WHI study as a whole, including a much larger sample than the one used in our present study, suggests that depression or antidepressant use may increase the vulnerability of postmenopausal women to age-related health problems can increase risks for frailty [7], all-cause and cause-specific mortality [18–20]. Thus, our present sub-study may be underpowered to detect an association between EDS and mortality as compared to the larger study.

Several studies have investigated antidepressant use and mortality. The results are mixed with two showing lower and one showing higher mortality in treated patients. Acharya et al. found that anti-depressant medication treatment lowered all-cause mortality in a sample of veterans especially among those with increased cardiovascular risk [70]. Qian et al. examined how depression diagnosis and antidepressant use was associated with mortality in a sample of young Social Security Disability Insurance recipients [71]. They concluded that antidepressant treatment lowered mortality significantly [71]. Hansen et al. investigated second-generation antidepressants and concluded that in their fully adjusted models, antidepressant use could slightly increase all-cause mortality [72].

Finally, our study showed that GrimAge is a potential mediator explaining the association between antidepressant use and all-cause mortality, although only before we adjusted for lifestyle and health-related factors. Given that GrimAge was conceived of as an independent predictor for all-cause mortality risk, this finding is consistent with our hypothesis, and shows that this clock is specifically explaining some of the TE of antidepressant use on mortality. Furthermore, it has recently been argued that GrimAge was developed as a measure of healthspan/lifespan [73]. Therefore, it may be more sensitive to capturing the impacts of the environment than DNAm predictors of chronological age. As people age, their DNAm predictors (e.g. Horvath, Hannum clocks) become more tightly coded and less susceptible to environmental inputs [73]. Future studies should examine which parts of this clock may be contributing to this mediating effect.

### Strengths and limitations

A strength of the study is that detailed data were collected at enrollment on all WHI participants, facilitating the evaluation of hypothesized relationships while taking key confounders into consideration. Second, it is possible to generalize study findings to postmenopausal women of diverse racial/ethnic backgrounds that reside in various geographical areas within the U.S.

However, there are several limitations of our study. First, ancillary study data were analyzed using a subsample of the original WHI participants, with missing data on exposure, mediator, moderator, outcome, and covariate variables potentially resulting in selection bias. In fact, the WHI includes participants who were recruited in a voluntary basis. It is not representative of all postmenopausal women in the U.S. Since there was no complex sampling design, and no weights in the original WHI study, there was no need to re-weight the analyses for this particular study which is based on a sub-sample of WHI. Third, measurement error is a notable issue when variables are assessed by self-report. Specifically, EDS was determined using a threshold on a screening instrument rather than a full diagnosis of depression and its severity. Therefore, it is still possible that use of antidepressants is a marker of severity of depression independently of EDS. Fourth, residual confounding due to unmeasured confounders as well as confounding by indication remain as concerns for observational study designs. Without repeated measurements of epigenetic age acceleration, depressive symptoms, and antidepressant use, reverse causality cannot be ruled out as an alternative explanation. Also, a causal relationship between epigenetic age acceleration, depressive symptoms, and antidepressant use, can only be definitively established in the context of an experimental design. Finally, the WHI is not population-based but involves volunteers at clinical centers, specifically targeting postmenopausal women. Therefore, its generalizability to men as well as younger and relatively less educated women is not possible.

## CONCLUSIONS

In summary, among postmenopausal women, higher GrimAge age acceleration partially explained the relationship between antidepressant use and increased all-cause mortality risk, though only prior to controlling for lifestyle and health-related factors. Antidepressant use and epigenetic age acceleration independently predicted increased all-cause mortality risk. Further studies are needed in comparable and diverse populations.

## Supplementary Materials

Supplementary Figure 1

Supplementary Table 1
