# Supplementary figures and images for: Relationships of depression and antidepressant use with epigenetic age acceleration and all-cause mortality among postmenopausal women
Source: Aging (Albany NY). 2024 May 27;16(10):8446–71. doi: 10.18632/aging.205868 (PMC11164525; doi:10.18632/aging.205868)

SUPPLEMENTARY FIGURE

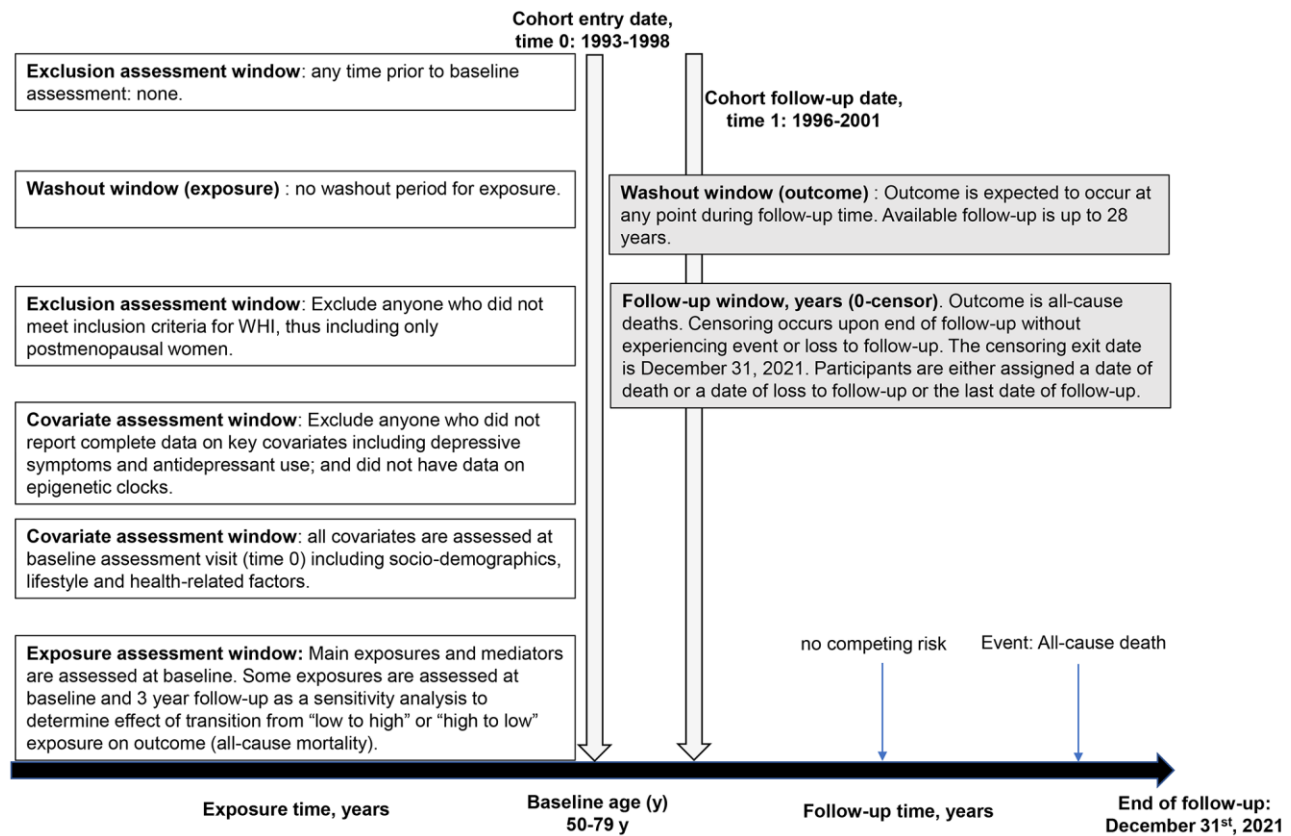

Supplementary Figure 1. Graphical depiction of the study design.

Supplement: Supplementary Figure 1 [file aging-16-205868-s001.pdf]
