# Supplementary material for: Relationships of depression and antidepressant use with epigenetic age acceleration and all-cause mortality among postmenopausal women
Source: Aging (Albany NY). 2024 May 27;16(10):8446–71. doi: 10.18632/aging.205868 (PMC11164525; doi:10.18632/aging.205868)
Supplement: Supplementary Table 1 [file aging-16-205868-s002.pdf]

SUPPLEMENTARY TABLE

Supplementary Table 1. Causal mediation analysis (four-way decomposition models) of the total effects of epigenetic age acceleration measures on mortality risk with baseline elevated depressive symptoms (EDS), antidepressant use (ANTIDEP) or combined exposure (EDS_ANTIDEP) as alternative mediators and/or moderators (*n* = 1,900).

X	M	Four-way decomposition parameter	MODEL 1			MODEL 2			MODEL 3		
			**β**	**SE**	***P***	**β**	**SE**	***P***	**β**	**SE**	***P***
IEAA	EDS	TE	0.027407	0.030382	0.367	***0.06012***	***0.03075***	***0.051***	0.042168	0.030107	0.161
IEAA	EDS	CDE	0.035034	0.03177	0.27	**0.071997**	**0.032002**	**0.024**	**0.053996**	**0.031653**	**0.088**
IEAA	EDS	INTREF	−0.00817	0.011681	0.484	−0.01217	0.01102	0.269	−0.01324	0.008621	0.125
IEAA	EDS	INTMED	0.000537	0.000921	0.56	0.000536	0.000938	0.567	0.000969	0.001073	0.367
IEAA	EDS	PIE	7.09E-06	0.000682	0.992	−2.44E-04	0.000571	0.669	4.40E-04	0.000734	0.549
EEAA	EDS	TE	**0.11742**	**0.033331**	**<0.001**	**0.129132**	**0.034021**	**<0.001**	**0.113847**	**0.033864**	**0.001**
EEAA	EDS	CDE	**0.118629**	**0.034891**	**0.001**	**0.135227**	**0.035315**	**<0.001**	**0.12437**	**0.035931**	**0.001**
EEAA	EDS	INTREF	−0.00093	0.012225	0.939	−0.00599	0.011984	0.617	−0.0098	0.009416	0.298
EEAA	EDS	INTMED	−0.00011	0.001445	0.939	−0.00049	0.001079	0.648	−0.00045	0.00084	0.591
EEAA	EDS	PIE	−0.00017	0.001231	0.891	0.000391	0.000895	0.662	−0.00027	0.00059	0.641
AgeAccelPheno	EDS	TE	**0.182011**	**0.035843**	**<0.001**	**0.204541**	**0.037093**	**<0.001**	**0.161381**	**0.035735**	**<0.001**
AgeAccelPheno	EDS	CDE	**0.180454**	**0.036805**	**<0.001**	**0.208895**	**0.037995**	**<0.001**	**0.166715**	**0.037244**	**<0.001**
AgeAccelPheno	EDS	INTREF	0.001544	0.0146	0.916	−0.00437	0.01418	0.758	−0.00549	0.011299	0.627
AgeAccelPheno	EDS	INTMED	0.000073	0.000699	0.917	−7.8E-05	0.000395	0.843	8.05E-05	0.00042	0.848
AgeAccelPheno	EDS	PIE	−5.9E-05	0.0005	0.906	9.41E-05	0.000404	0.816	0.00008	0.000408	0.844
AgeAccelGrim	EDS	TE	**0.29565**	**0.036358**	**<0.001**	**0.327529**	**0.040086**	**<0.001**	**0.265959**	**0.046626**	**<0.001**
AgeAccelGrim	EDS	CDE	**0.316078**	**0.039023**	**<0.001**	**0.339236**	**0.042165**	**<0.001**	**0.281169**	**0.048263**	**<0.001**
AgeAccelGrim	EDS	INTREF	−0.01662	0.011246	0.139	−0.01055	0.011877	0.374	−0.01472	0.009771	0.132
AgeAccelGrim	EDS	INTMED	−0.0031	0.002411	0.198	−0.00164	0.00201	0.414	−0.0004	0.001308	0.761
AgeAccelGrim	EDS	PIE	−0.0007	0.001953	0.719	0.000488	0.001585	0.758	−8.9E-05	0.000384	0.817
IEAA	ANTIDEP	TE	0.026303	0.03095	0.395	**0.062246**	**0.031143**	**0.046**	0.04036	0.03044	0.185
IEAA	ANTIDEP	CDE	0.027571	0.030524	0.366	**0.061114**	**0.030954**	**0.048**	0.043247	0.030582	0.157
IEAA	ANTIDEP	INTREF	−0.00359	0.011851	0.762	−0.00149	0.010693	0.889	−0.00438	0.009414	0.641
IEAA	ANTIDEP	INTMED	−0.0003	0.001045	0.773	−0.00012	0.000896	0.891	−0.00031	0.000785	0.69
IEAA	ANTIDEP	PIE	0.002623	0.002858	0.359	0.002745	0.003175	0.387	0.001811	0.002421	0.454
EEAA	ANTIDEP	TE	**0.128582**	**0.034868**	**<0.001**	**0.139916**	**0.035274**	**<0.001**	**0.112495**	**0.034025**	**0.001**
EEAA	ANTIDEP	CDE	**0.106216**	**0.033128**	**0.001**	**0.12211**	**0.033749**	**<0.001**	**0.108782**	**0.034044**	**0.001**
EEAA	ANTIDEP	INTREF	0.01743	0.014849	0.24	0.014681	0.014246	0.303	0.002495	0.01067	0.815
EEAA	ANTIDEP	INTMED	0.001828	0.002218	0.41	0.00097	0.001661	0.559	0.000112	0.000534	0.834
EEAA	ANTIDEP	PIE	0.003109	0.002902	0.284	0.002155	0.003117	0.489	0.001107	0.002326	0.634
AgeAccelGrim	ANTIDEP	TE	**0.178892**	**0.035431**	**<0.001**	**0.205059**	**0.036746**	**<0.001**	**0.162047**	**0.035753**	**<0.001**
AgeAccelGrim	ANTIDEP	CDE	**0.184241**	**0.036073**	**<0.001**	**0.207841**	**0.037352**	**<0.001**	**0.167278**	**0.036557**	**<0.001**
AgeAccelGrim	ANTIDEP	INTREF	−0.00467	0.011651	0.689	−0.00208	0.011179	0.852	−0.00379	0.009531	0.691
AgeAccelGrim	ANTIDEP	INTMED	0.000119	0.000481	0.805	4.57E-05	0.000304	0.881	0.000248	0.000704	0.725
AgeAccelGrim	ANTIDEP	PIE	−0.0008	0.00256	0.754	−0.00074	0.002935	0.8	−0.00169	0.00228	0.458
AgeAccelGrim	ANTIDEP	TE	**0.296287**	**0.036399**	**<0.001**	**0.330774**	**0.040302**	**<0.001**	**0.26638**	**0.046889**	**<0.001**
AgeAccelGrim	ANTIDEP	CDE	**0.286615**	**0.037212**	**<0.001**	**0.30887**	**0.040733**	**<0.001**	**0.260132**	**0.046933**	**<0.001**
AgeAccelGrim	ANTIDEP	INTREF	0.004317	0.010551	0.682	0.01278	0.011209	0.254	0.002445	0.009382	0.794
AgeAccelGrim	ANTIDEP	INTMED	0.00074	0.001848	0.689	0.002878	0.002815	0.307	0.000348	0.00138	0.801

AgeAccelGrim	ANTIDEP	PIE	0.004616	0.003005	0.124	0.006246	0.003503	0.075	0.003455	0.003124	0.269
IEAA	EDS_ANTIDEP	TE	0.024951	0.030867	0.419	***0.05628***	***0.03108***	***0.07***	0.037564	0.030254	0.214
IEAA	EDS_ANTIDEP	CDE	0.034332	0.031718	0.279	**0.07147**	**0.032069**	**0.026**	***0.05517***	***0.03191***	***0.084***
IEAA	EDS_ANTIDEP	INTREF	−0.00942	0.016635	0.571	−0.01548	0.015928	0.331	−0.01757	0.013783	0.202
IEAA	EDS_ANTIDEP	INTMED	−1.6E-05	0.000487	0.974	−0.00018	0.000855	0.832	0.000175	0.000962	0.856
IEAA	EDS_ANTIDEP	PIE	5.76E-05	0.001767	0.974	0.000472	0.002178	0.828	−0.00021	0.001168	0.855
EEAA	EDS_ANTIDEP	TE	**0.118092**	**0.033739**	**<0.001**	**0.127094**	**0.034381**	**<0.001**	**0.109143**	**0.033757**	**0.001**
EEAA	EDS_ANTIDEP	CDE	**0.113213**	**0.034885**	**0.001**	**0.13155**	**0.035283**	**<0.001**	**0.124907**	**0.036301**	**0.001**
EEAA	EDS_ANTIDEP	INTREF	0.002157	0.017292	0.901	−0.00579	0.017534	0.741	−0.01582	0.014508	0.276
EEAA	EDS_ANTIDEP	INTMED	0.000169	0.001361	0.901	−0.00023	0.000765	0.765	−0.00017	0.000901	0.846
EEAA	EDS_ANTIDEP	PIE	0.002552	0.002113	0.227	0.001559	0.002269	0.492	0.000231	0.00118	0.845
AgeAccelGrim	EDS_ANTIDEP	TE	**0.178451**	**0.035762**	**<0.001**	**0.20038**	**0.036953**	**<0.001**	**0.160073**	**0.03576**	**<0.001**
AgeAccelGrim	EDS_ANTIDEP	CDE	**0.183306**	**0.037339**	**<0.001**	**0.213135**	**0.038619**	**<0.001**	**0.174037**	**0.03809**	**<0.001**
AgeAccelGrim	EDS_ANTIDEP	INTREF	−0.0051	0.018362	0.781	−0.01258	0.018163	0.489	−0.01358	0.015672	0.386
AgeAccelGrim	EDS_ANTIDEP	INTMED	−4.4E-05	0.000308	0.887	8.09E-05	0.000681	0.905	0.000586	0.000997	0.557
AgeAccelGrim	EDS_ANTIDEP	PIE	0.000291	0.001762	0.869	−0.00026	0.002156	0.904	−0.00097	0.001334	0.469
AgeAccelGrim	EDS_ANTIDEP	TE	**0.293954**	**0.036326**	**<0.001**	**0.326586**	**0.040187**	**<0.001**	**0.264104**	**0.046683**	**<0.001**
AgeAccelGrim	EDS_ANTIDEP	CDE	**0.303576**	**0.039729**	**<0.001**	**0.3254**	**0.042934**	**<0.001**	**0.282577**	**0.048697**	**<0.001**
AgeAccelGrim	EDS_ANTIDEP	INTREF	−0.01186	0.01562	0.448	−0.00329	0.01692	0.846	−0.01859	0.014623	0.204
AgeAccelGrim	EDS_ANTIDEP	INTMED	−0.00175	0.002395	0.465	−0.0005	0.002567	0.846	-0.00047	0.001317	0.721
AgeAccelGrim	EDS_ANTIDEP	PIE	0.003991	0.002595	0.124	***0.00498***	***0.0029***	***0.086***	0.000593	0.001632	0.717

Abbreviations: AgeAccel GrimAge: GrimAge epigenetic age acceleration; AgeAccel Pheno: PhenoAge epigenetic age acceleration; ANTIDEP: Antidepressant use; CDE: Controlled Direct Effect; EDS: Elevated Depressive Symptoms; EDS_ANTIDEP: Either EDS or ANTIDEP; EEAA: Extrinsic Epigenetic Age Acceleration; HR: Hazard Ratio; IEAA: Intrinsic Epigenetic Age Acceleration; INTMED: Mediated Interaction; INTREF: Interaction Referent; PIE: Pure Indirect Effect; SE: Standard Error; TE: Total Effect; X: Exposure. Values are estimates ± SE from four-way decomposition models and their *p*-values, with final equation being a Cox PH model for all-cause mortality, mediators being each of the EDS and antidepressant use baseline mediators; and exposures being each of the epigenetic clock metrics. Model 1 is unadjusted; Model 2 adjusted for sociodemographic; Model 3 is Model 2 further adjusted for lifestyle and health characteristics. P is for null hypothesis that $\beta = 0$. Bolded values are when $P < 0.05$.
